# Development and Bio-Predictive Evaluation of Biopharmaceutical Properties of Sustained-Release Tablets with a Novel GPR40 Agonist for a First-in-Human Clinical Trial

**DOI:** 10.3390/pharmaceutics13060804

**Published:** 2021-05-28

**Authors:** Ewelina Juszczyk, Kamil Kisło, Paweł Żero, Ewa Tratkiewicz, Maciej Wieczorek, Jadwiga Paszkowska, Grzegorz Banach, Marcela Wiater, Dagmara Hoc, Grzegorz Garbacz, Jaroslaw Sczodrok, Dorota Danielak

**Affiliations:** 1 Research and Development Center, Celon Pharma S.A., Marymoncka 15, 05-052 Kazuń Nowy, Poland; ewelina.juszczyk@celonpharma.com (E.J.); kamil.kislo@celonpharma.com (K.K.); pawel.zero@celonpharma.com (P.Ż.); ewa.tratkiewicz@celonpharma.com (E.T.); maciej.wieczorek@celonpharma.com (M.W.); 2 Physiolution Polska sp. z o.o., 74 Piłsudskiego St., 50-020 Wrocław, Poland; j.paszkowska@physiolution.pl (J.P.); g.banach@physiolution.pl (G.B.); m.wiater@physiolution.pl (M.W.); d.hoc@physiolution.pl (D.H.); g.garbacz@physiolution.pl (G.G.); 3 Physiolution GmbH, Walther Rathenau Strasse 49a, 17489 Greifswald, Germany; jsczodrok@physiolution.eu; 4 Department of Physical Pharmacy and Pharmacokinetics, Poznan University of Medical Sciences, 6 Święcickiego St., 60-781 Poznań, Poland

**Keywords:** GPR40 agonist, sustained-release tablets, first in human clinical trials, simulation of gastrointestinal passage, biorelevant dissolution testing, stress test device

## Abstract

Sustained-release (SR) formulations may appear advantageous in first-in-human (FIH) study of innovative medicines. The newly developed SR matrix tablets require prolonged maintenance of API concentration in plasma and should be reliably assessed for the risk of uncontrolled release of the drug. In the present study, we describe the development of a robust SR matrix tablet with a novel G-protein-coupled receptor 40 (GPR40) agonist for first-in-human studies and introduce a general workflow for the successful development of SR formulations for innovative APIs. The hydrophilic matrix tablets containing the labeled API dose of 5, 30, or 120 mg were evaluated with several methods: standard USP II dissolution, bio-predictive dissolution tests, and the texture and matrix formation analysis. The standard dissolution tests allowed preselection of the prototypes with the targeted dissolution rate, while the subsequent studies in physiologically relevant conditions revealed unwanted and potentially harmful effects, such as dose dumping under an increased mechanical agitation. The developed formulations were exceptionally robust toward the mechanical and physicochemical conditions of the bio-predictive tests and assured a comparable drug delivery rate regardless of the prandial state and dose labeled. In conclusion, the introduced development strategy, when implemented into the development cycle of SR formulations with innovative APIs, may allow not only to reduce the risk of formulation-related failure of phase I clinical trial but also effectively and timely provide safe and reliable medicines for patients in the trial and their further therapy.

## 1. Introduction

Maintaining the drug concentration within a specific therapeutic range is a crucial factor in chronic diseases, such as diabetes mellitus type 2 (T2DM). To better control glycemia, several of the most commonly prescribed oral antidiabetic agents are available as sustained-release (SR) oral dosage forms. Such systems provide several key advantages that make them an asset in the therapeutic management of T2DM.

For commonly used drugs, such as metformin or sulfonylurea derivatives, an SR formulation offers the same clinical benefits but with a reduced dosage, fewer side effects, and better patient satisfaction with the treatment compared with a standard dosage form [1,2,3,4]. Better patient compliance and lower risk of hypoglycemia make SR solid dosage forms a useful tool in the treatment of T2DM. Another benefit of SR formulations is their usefulness for drugs with a short biological half-life [5].

As a result of the reasons mentioned above, an SR formulation may appear advantageous for new antidiabetic drugs entering a clinical trial in humans, with a special case of oral dosage forms for the first-in-human (FIH) study of innovative medicines. In such cases, the determination of a simple oral solution or an immediate release capsule is not necessarily recommended. It holds true, especially if the candidate drug undergoes a rapid elimination and a long-lasting exposition is required for the clinically desired pharmacodynamic effect. A simple and robust SR product with the desired delivery rate offers a desired in vivo efficacy and reliable bioavailability estimation. In these circumstances, introducing this type of formulation should be considered and launched as early as possible. Several types of such systems are currently available on the market. SR solid dosage forms include matrix or coated tablets, multiparticulate drug delivery systems, 3D-printed tablets, or osmotically controlled drug delivery systems [6]. The last system, OROS, offers a unique zero-order kinetic release rate that is pH-independent [7]. However, the OROS technology is laborious in the development and difficult to manufacture, and owing to its non-dispersibility, it may be problematic in gastrointestinal (GI) narrowings or motility disorders [8]. Therefore, monolithic matrix tablets are still widely used as simple, robust, and easy to manufacture SR delivery systems.

A thorough characterization of the newly developed SR matrix tablet should reliably assess the risk of uncontrolled drug release. Several critical factors modulate drug dissolution from hydrophilic matrices [9]. First, there are the properties of the drug, such as solubility, dissociation constant, stability, or molecular weight. Second, there are the type, properties, and particle size of the API and polymer excipients used in the matrix. Third, the formulation factors, such as the geometry of the matrix, ionic strength, or microenvironmental pH, may alter the pH-dependent solubility [10]. The SR tablet developed without considering these factors may be prone to a burst effect when a large amount of drug is released in contact with a medium or to dose dumping when the release of the drug is accelerated. Evaluation of the dissolution characteristics should reveal these unwanted and potentially threatening performances. Conventional USP compliant dissolution tests offer a quick and straightforward characterization but may lack a discriminative power to detect the impact of biorelevant media mimicking GI fluids or mechanical stress observed during GI passage [11,12,13].

In the present study, we describe the development of a robust SR matrix tablet with a novel G-protein-coupled receptor 40 (GPR40) agonist for first-in-human studies. The main aim of this paper is to propose a general workflow for the successful development of a novel SR tablet with an innovative API. The proposed step-by-step approach focuses on employing various analytical methods, including tablet swelling and water uptake analysis, through standard USP compliant dissolution tests, and sophisticated physiology-driven biorelevant dissolution tests. This novel pathway may be useful for the effective and reliable characterization of a robust SR formulation and offers a reliable prediction of in vivo pharmacokinetics.

## 2. Materials and Methods

### 2.1. Basic Characteristics of the Formulations

The active pharmaceutical ingredient (API), labeled CPL207-280-51, is an innovative BCS class I compound. This new GPR40 agonist exhibits antidiabetic properties and is intended to enter a first-in-human clinical trial. To date, there is a lack of data on the pharmacokinetics of this API in humans.

The formulation process aimed to develop three strengths of SR tablets suitable for dose-escalating studies in phase I clinical trials. The tablets should resist the GI passage under both fasted and fed conditions, without the risk of dose dumping. The dosage form should release the API gradually for up to 14–18 h, regardless of the tablet strength, to allow a once-daily administration.

The hydrophilic matrix tablets containing the labeled API dose of 5, 30, or 120 mg were manufactured by direct compression. First, the API and the excipients were mixed. Then, the mixture was compressed using an XP-1 single-punch tablet press (Korsch, Berlin, Germany) and EUD oval punch, 19 × 9 mm. The final tablets’ weights and dimensions were kept fixed for all tablet strengths to assure a constant surface to volume ratio [14]. It was suspected that the overall matrix-forming ability might change upon increasing API dose because of modifying the total matrix solubility (the API is a well-soluble compound). It could result in alterations in dissolution profiles between the tablet strengths. Hence, the excipients used in the formulation were divided into two groups: matrix-forming and matrix-filling ingredients. The matrix-forming agents assured a sustained release of the API, and the fillers were responsible for tablet compression. The amount of one of the matrix formers was reduced upon increasing the API labeled dose so that the proportion between matrix formers and matrix fillers was constant regardless of the API amount. Then, total matrix solubility was established on a suitable level. All excipients were of pharmaceutical grade.

In order to search for the possible interactions between the tested compound and various excipients used in pharmaceutical drug development, an incompatibility test was designed. The binary mixtures of the API and selected excipients were stored in both open and closed glass vials. The samples were stored for 4 weeks at 40 °C and 75% relative humidity. The amount of the API was analyzed using HPLC and compared with the initial value. The following excipients were evaluated: lactose monohydrate, microcrystalline cellulose (MCC), siliconized microcrystalline cellulose (SMCC), mannitol, calcium hydrogen phosphate dihydrate, dicalcium phosphate anhydrous, disodium phosphate, pregelatinised starch, Neusilin, povidone (PVP), hypromellose (HPMC), crospovidone, copovidone, crosscarmellose sodium (CCS), silicon dioxide, and magnesium stearate.

### 2.2. Analytical Methods and Dissolution Tests Setup

#### 2.2.1. Standard Dissolution Testing

##### Test Conditions

Standard dissolution tests were performed using the USP type II paddle apparatus set at 75 rpm and 37 °C. The dissolution medium was 900 mL USP phosphate buffer pH = 6.8. The samples were collected at time points of 0.5, 1, 2, 3, 5, 8, 12, 18, and 18.25 h; then, they were filtered through a 0.45 µm cellulose syringe filter. All tests were performed for at least *n* = 3 tablets.

##### Determination of the API in Standard Dissolution Media

Collected samples were analyzed with the high-performance liquid chromatography (HPLC) method, which was validated according to the International Council for Harmonisation of Technical Requirements for Pharmaceuticals for Human Use (ICH) guidelines [15]. Five µL (for 30 mg and 120 mg tablet strengths) or 20 µL (for 5 mg tablet strength) was injected onto a Zorbax Eclipse Plus Phenyl-Hexyl column (3.0 × 150 mm, 3.5 µm, Agilent, Santa Clara, CA, USA) equipped with an inline filter. Gradient elution at a 1 mL/min flow rate was employed, and two mobile phases were used—phase A consisted of 0.1% phosphoric acid; phase B was pure acetonitrile. Initial conditions were 70% phase A: 30% phase B. After 6 min, the amount of phase A decreased to 50%; then, after 12 min, it decreased to 45%, and from 13 to 14.5 min, it decreased to 5%. After 14.5 min, the column was re-equilibrated to the initial conditions. The total run time of the method was 15 min. A UV-VIS detection was performed at λ = 275 nm. The method was linear within 1.4–140 mg/L, with the lower limits of quantitation of the calibration curve: 1.4 mg/L for the low-strength (5 mg API), 7.0 mg/L for medium-strength (30 mg), and 56 mg/L for the high-strength (120 mg) batches.

#### 2.2.2. Prototype Performance Evaluation

##### Water Sorption and Swelling Kinetics Analysis

The tested formulations were incubated in 0.1 M HCl, 50 mM potassium phosphate buffer pH = 3.0 with 0.1% Tween 80, and in the medium simulating intestinal fluids under fasted conditions (FaSSIF). FaSSIF comprised 0.420 g sodium hydroxide, 4.470 g sodium hydrogen phosphate dihydrate, and 6.186 g sodium chloride dissolved in 900 mL demineralized water. After the components were fully dissolved, the pH was adjusted to 6.5 with 1 M sodium hydroxide, and the volume was made up to 1000 mL with water. Into the solution, 2.240 g of FaSSIF/FeSSIF (Fed State Simulated Intestinal Fluid)/FaSSGF (Fasted State Simulated Gastric Fluid) powder (Biorelevant.com Ltd., London, UK) was added, and the medium was stirred until the powder fully dissolved. For better visualization of the hydration front, a colorant was added into both the tablet prototypes and incubation media at a concentration of approximately 0.2%. All of the reagents were of analytical grade.

The tested tablets were weighed before incubation, and their dimensions were measured. They were incubated in 250 mL of preheated medium (37 °C) for 2 or 5 h, depending on the solution. Samples were withdrawn from the solution every hour, measured, and photographed (Supplementary Figure S1). After incubation, the tablets were weighed again. Then the tablets were cut with a scalpel, and their cross-sections were photographed and measured.

##### Texture Kinetics

The tablets were hydrated by incubation in beakers in 40 mL of simulated gastric fluid (SGF) pH = 1.2, in the 50 mM phosphate buffer pH = 3.0 with 0.1% Tween 80, and in FaSSIF for 2 h at 37 °C. They were also incubated for 5 h in beakers with 40 mL of phosphate buffer pH = 3.0 with 0.1% Tween 80 and in FaSSIF at 37 °C. The tablets were placed on a piece of cellulose paper to prevent adhesion to the walls of the beaker. The tablets were tested using a texture analyzer TA.XTplus Texture Analyser (Stable Microsystems, Surrey, UK) equipped with a 50 N load cell and a pushrod diameter of 35 mm. The hydrated tablets were withdrawn from the incubation medium and placed on a piece of aluminum foil on the lower plastic plate, and the test was initiated at a crosshead speed of 0.5 mm/min. The trigger force equaled 0.05 N. The load was directed perpendicularly to the long axis of the tablet. The measurement duration was 150 s at a sampling time of 0.025 s. A visual inspection of the tablets was also recorded.

#### 2.2.3. Biorelevant Dissolution Tests

Biorelevant dissolution tests were performed in the StressTest^®^ device, which was first introduced by G. Garbacz and W. Weitschies [11]. The apparatus simulates the mechanical agitation of physiological intensity. In combination with the biorelevant media, the equipment mimics the physiological conditions that act on a solid dosage form in subsequent sections of the GI tract. A detailed description of the device is given elsewhere [16].

##### Biorelevant Test Protocols

The detailed setup of all biorelevant test protocols is presented in Table 1. All of the tests were performed for *n* = 3–6 tablets. For simulated fasted conditions, four test programs were used, reflecting standard gastric emptying, an acidified gastric environment, increased mechanical agitation in the GI tract, and delayed gastric emptying. For simulated fed conditions, three test programs were applied: regular gastric emptying, regular gastric emptying in whole milk containing digestive enzymes, and increased gastric mechanical agitation. The detailed composition of the media is listed below in a separate section.

Sampling was performed according to the chosen API determination method. Detailed information on the determination methods is given below. All samples were filtered through 1 µm poroplast filters (Dissolution Accessories, Oosterhout, The Netherlands). For UV-VIS measurements, sampling was performed in a closed-loop system. Within the first three hours, the samples were taken every 5 min, and then every 10 min until the end of the test. For HPLC measurements, 1 mL aliquots were sampled automatically at the predefined time points: 0 h, 0.5 h, every hour until 21 h, and at 22.5 and 24 h after the start of the experiment. The samples were transferred as soon as possible into an HPLC autosampler with a thermostat set at 12 °C or ambient temperature.

Sample taking from whole milk containing digestive enzymes was conducted manually for up to 12 h, in which samples of 0.4 mL obtained from milk were mixed in a volumetric ratio of 1:3 with cold acetonitrile (1.2 mL), thoroughly vortexed for 5 s, and centrifuged for 10 min at 24,000× *g*. Then, 600 µL of the supernatant was transferred to HPLC vials, capped, and immediately transferred to a cooled HPLC autosampler.

##### Media

The composition of the media was adjusted according to the test protocols specified in Table 1. To simulate intragastric fluids under standard fasted conditions, a USP SGF sine pepsin pH = 1.8 was used. Intestinal fluids under fasted conditions were simulated using a FaSSIF/FeSSIF/FaSSGF powder concentrate dissolved in 50 mM potassium phosphate buffer (pH = 6.8) in concentration of 2.24 g/L. In the modified fasted protocols, the pH of SGF was set to 1.2 for the acidified gastric environment or to 1.8 for increased mechanical agitation protocol and delayed gastric emptying protocol. Intestinal fluids were simulated as described above.

Intragastric fluids under fed conditions were simulated using a 50 mM phosphate buffer of an initial pH = 4.5. The pH of the buffer was gradually decreased to 2.0, according to the applied test protocol (Table 1). Intestinal fluids under fed conditions were simulated by adding 40 mL of a FaSSIF/FeSSIF/FaSSGF powder pre-dissolved in 50 mM potassium phosphate buffer (77.9 g dissolved in 240 mL of the buffer) to each of the test vessels. The final concentration of FaSSIF/FeSSIF/FaSSGF in the test vessels was 11.2 g/L. Immediately after the addition of the concentrate, the pH was adjusted to 6.5 with a 40% sodium hydroxide solution.

In modified tests under simulated fed conditions, in which the media comprised whole milk (3.5% fat), acidification was performed similarly as in the phosphate buffer, with the gradual addition of pepsin (Merck, Darmstadt, Germany) up to the final concentration of 2.0 g/L. The intestinal transition was simulated with the addition of FaSSIF/FeSSIF/FaSSGF powder as described above and containing pancreatin (SAFC, Hamburg, Germany) to reach a final concentration of 4.57 g/L.

##### Determination of the API in the Media

Determination of the API in the dissolution media was performed with a UV-VIS method and two HPLC methods, labeled ‘A’ and ‘B’. All methods were validated according to ICH guidelines [14].

The UV-VIS spectroscopy method was performed with an Agilent 8543 spectrophotometric system (Agilent, Santa Clara, CA, USA). The flow-through quartz cuvettes were used, of a 10 mm path length (Hellma, Müllheim, Germany). The photometer was set to a single wavelength reference mode with a λ = 275 nm for API determination and λ = 295 nm as the reference. The method was linear within a 15–150 mg/L concentration range.

HPLC method A was used to determine the API in the samples collected during tests in simulated fed conditions and determine the API in experiments involving the batches containing the lowest amount (5 mg) of the API. The resolution was obtained on a Zorbax Eclipse Plus Phenyl-Hexyl column (3.0 × 150 mm, 3.5 µm, Agilent) equipped with an inline filter and maintained at 35 °C. Gradient elution at a 1 mL/min flow rate was employed, and two mobile phases were used—A, which consisted of 0.1% phosphoric acid/acetonitrile (60:40, *v*:*v*); B, which consisted of 0.1% phosphoric acid/acetonitrile (10:90, *v*:*v*). During the first 12 min, 100% of mobile phase A was supplied. Then, the mobile phase was switched to 100% B to wash out impurities from the media. At 15.5 min post-injection, the column was re-equilibrated to the initial conditions with the mobile phase A. The total run time of the method was 22 min. The determination was performed on a fluorescence detector, with excitation wavelength λ = 220 nm, and emission λ = 309 nm and simultaneously on a UV-VIS detector set to λ = 275 nm. The quantitation limit of the fluorescence method was 0.49 mg/L, while for the UV-VIS detection, it was 2.01 mg/L. Both detection methods were linear up to 144 mg/L. The Bland–Altman analysis [17] showed that the two detection methods are in agreement for the concentrations of 5 mg/L or higher. For the lower concentrations, only the fluorescence detection method was employed.

The HPLC method B was used for the determination of the API in samples collected in experiments under simulated fasted conditions. The resolution was performed on a Waters Symmetry RP18 column (3.9 × 20 mm, 3.5 µm, Waters) with an inline filter and maintained at 30 °C. Then, 10 µL sample volume was injected into the chromatographic system. Isocratic conditions were used with 0.1% phosphoric acid/acetonitrile (60:40, *v*:*v*) as the mobile phase. The total runtime of the method was 2.5 min. The detection wavelength of the UV-VIS detector was 275 nm. The quantitation limit was 2.01 mg/L, which was calculated from the ratio of the standard deviation of blank sample responses and the slope of the calibration curve, and the method was linear up to 144 mg/L. To assure complete elution of impurities that could have been retained on the column, a cleaning procedure was performed every six injections. It involved a 10-min long elution, which involved a gradual introduction of a more acetonitrile-rich mobile phase (0.1% phosphoric acid/acetonitrile, 10:90, *v*:*v*). During the cleaning procedure, the amount of the acetonitrile-rich phase increased from 0 to 15% from 0.2 to 5.5 min. After this time, the column was re-equilibrated to the initial conditions.

### 2.3. Experiment Workflow

**Figure 1:**
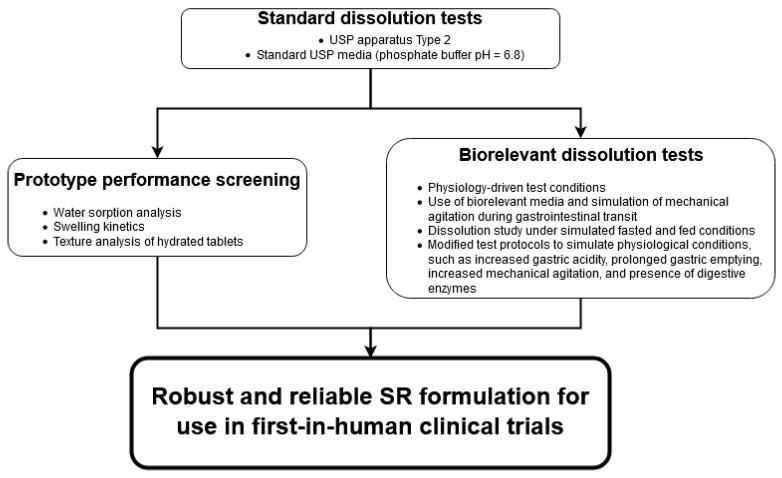
The analysis workflow.

The general workflow of the study is presented in Figure 1.

First, the standard USP tests were performed to evaluate the basic dissolution profile under standard conditions rapidly. The aim was to verify that the API was released in an extended way within a 20 h time frame. The best performing formulations were selected for further in vitro performance evaluation and biorelevant dissolution tests.

Second, the performance of the prototype tablets was evaluated. This stage aimed to exclude the formulations that disintegrated during incubation in media simulating gastric conditions. Leading formulations were characterized by good mechanical stability and integrity.

In parallel, during the biorelevant dissolution tests in the StressTest device, the formulations were screened for the risk of potential dose dumping under various physiology-oriented conditions. The formulations that did not show an undesired drug release upon mechanical agitation and/or media change and demonstrated similar performance regardless of the API dose were selected for clinical studies.

## 3. Results

### 3.1. Standard USP Dissolution Tests

The analysis of the API degradation in the presence of various excipients showed that none of the tested substances significantly influenced API stability. Therefore, the excipients could be safely used for manufacturing prototypes and final formulations. The compositions of all the developed batches are presented in the Supplementary Table S1.

**Figure 2:**
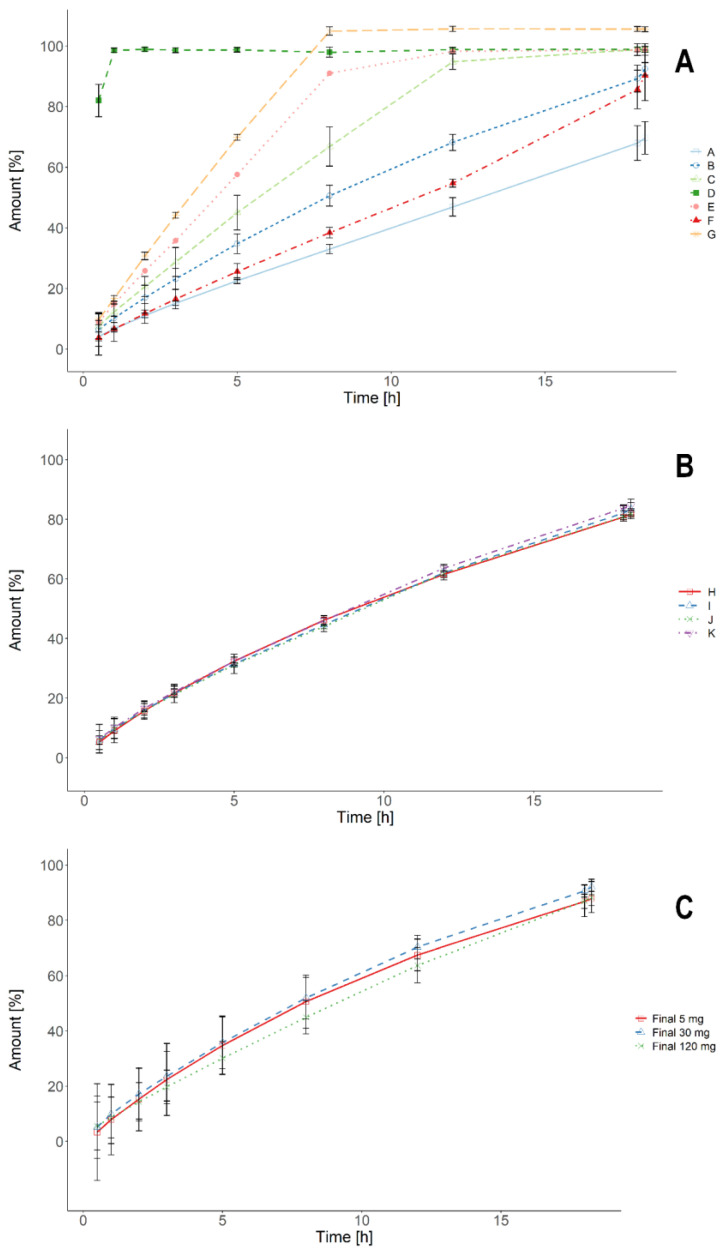
The dissolution profiles obtained in the USP II paddle apparatus in phosphate buffer pH = 6.8. Profiles for prototypes (Panels **A** and **B**) are presented as means from *n* = 3 replicates, while final formulations of hardness 185–225 N (Panel **C**) are presented as means from *n* = 6 replicates, and standard deviations as error bars.

After selecting the excipients compatible with the API, the prototype formulations were tested in the USP type II paddle apparatus (Figure 2).

Figure 2A shows the dissolution profiles obtained for initially prepared formulations (A–G). The formulations varied greatly in their dissolution characteristics, ranging from an almost immediate-release (D) to a very extended one (A). The optimization of excipients’ concentrations allowed obtaining the desired dissolution rate (Figure 2B). All the manufactured prototypes (H–K) had very similar dissolution kinetics. To further characterize the prototypes and choose the leading formulation, the next steps of the development process employed varied analytical techniques involving biorelevant media and physiologically based dissolution.

### 3.2. Preformulation Analysis

#### 3.2.1. Water Sorption Analysis

Table 2 presents the results of the water sorption analysis for the prototype formulation and the final tablets of different strengths and hardness 185–225 N. All of the tested batches increased their mass by approximately 100% after 2 h incubation in 0.1 M HCl. However, the transition to the phosphate buffer and FaSSIF resulted in up to a three-fold increase in the tablet mass. The water uptake did not differ greatly between tablets incubated in these two media, regardless of the tablet composition. The prototype (J) absorbed more water than the final formulations in all of the media tested. All strengths of the final formulations behaved similarly, showing robustness and good integrity.

#### 3.2.2. Swelling Kinetics

Table 2 shows the change in the tablet dimensions and the dry area fraction after incubation in different media. The visual observation of the tablets showed that all of the tested tablets increased mainly their relative height, whereas their relative width changed only slightly. The dimensions of the prototype increased to the greatest extent in comparison with the final formulations.

Visualization of the movement of the erosion and swelling fronts was possible due to the presence of a colorant. According to Colombo et al. [18], the erosion front is separating the matrix from the solvent, while the swelling front separates the rubbery and glassy regions in the matrix. The dissection of the tablet revealed the dry part, which remained white, while the colorant marked the colored hydrated fraction. The prototype batch (J) was the one in which the swelling front moved inwards toward the core to the greatest extent. As little as 27% of the total area of the tablet cross-section remained dry. The final batches (hardness 185–225 N) were more resistant than the prototype, especially in 0.1 M HCl. In this medium, more than 50% of the tablet remained dry. In both phosphate buffer and FeSSIF, the fraction of the tablet cross-section that remained dry was approximately 40% for all the strengths.

#### 3.2.3. Texture Analysis

**Figure 3:**
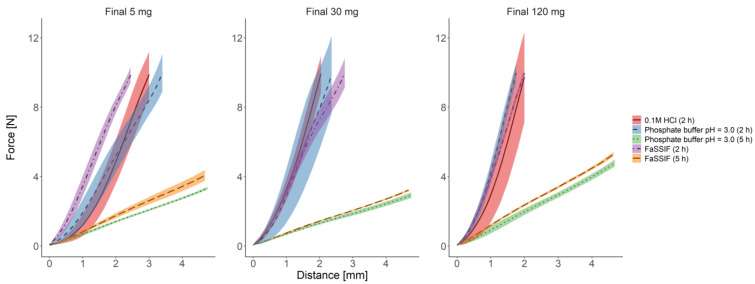
Mean deformation profiles obtained for the final formulations of hardness 185–225 N (*n* = 3) with standard deviations presented as shaded areas.

Figure 3 presents the deformation profiles obtained after measuring the probe penetration depth into the swollen tablets, which was incubated in various media. Due to the destructive nature of the study, the texture analysis was performed only for the final batches with the hardness of 185–225 N.

After incubating the tested batches for 2 h in all media, the formulations showed high resistance to deformation, regardless of the API dose. It indicates that in all tested media, the tablets contained a dry core. The high variability of deformation profiles resulted from the small differences in the water uptake and little propagation distances of the pushrod. It was observed especially in the measurements performed at 2 h due to low water penetration into the tablets (<2 mm penetration depth).

Fluctuations of deformation profiles decreased after a 5 h incubation in phosphate buffer pH 3.0 and FaSSIF. It suggests that the hydrogel layers surrounding the dry tablet core were stable and withheld their mechanical integrity despite higher swelling and hydration. After prolonged incubation, crossing the eroded part of the tablet required the highest force for the formulation containing 120 mg API. It indicates that the mechanical robustness of the formulation may increase with the API dose.

### 3.3. Biorelevant Dissolution Tests

**Figure 4:**
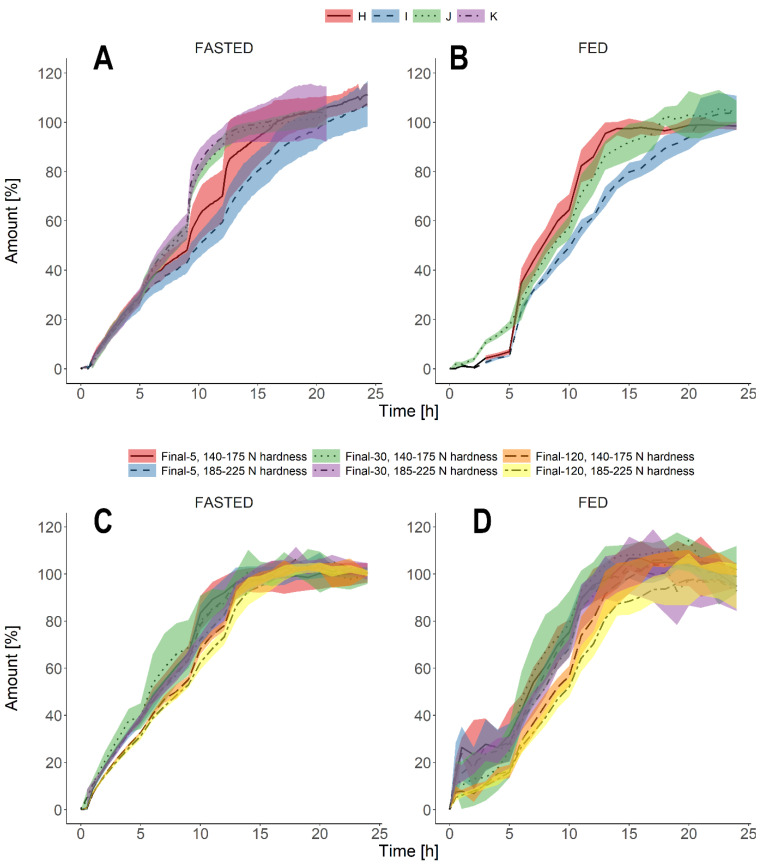
Comparison of dissolution profiles obtained in biorelevant media in the StressTest device under simulated standard fasted and fed conditions. (**A**,**B**) Dissolution profiles of the prototypes. (**C**,**D**) Dissolution profiles of the final formulations of different hardnesses, containing 5, 30, and 120 mg of the API. The data are presented as means of the cumulative amount of the drug released in percent (*n* = 6 for final, J and K batches, *n* = 3 for batches H and I under simulated fasted conditions, *n* = 2 for batches H and I under simulated fed conditions) with standard deviations presented as shaded areas.

According to the study protocol, the prototype batches that fulfilled the initial criteria were investigated in a biorelevant dissolution test, including physiology-driven mechanical agitation patterns. Figure 4A depicts the dissolution profiles of the prototypes (H, I, J, K) under simulated fasted conditions, as presented in Table 1. Mechanical agitation during this experiment caused an extensive release of the API into the media for almost all of the tested batches, which may cause dose-dumping episodes in vivo. The amount of the API released into the media increased to the greatest extent in the ninth hour of the experiment, when the StressTest device simulated the passage through the colon. The increase was most pronounced for batches J and K, which released almost 30% of the total amount of the API within a very short time. Batch H had two occurrences of intensified API release in the ninth and twelfth hour of the experiment. Batch I was the most durable and resistant to programmed stress events. However, the release profiles of this batch, as well as of batch H, had considerably high variability.

Batches H, I, and J were tested under simulated fed conditions (Figure 4B); dissolution from batch K was not assessed due to resemblance to batch J. Batches H and I resisted both gradually increasing acidity and mechanical agitation during the first five hours of the experiment when intragastric conditions were simulated. After adding bile salts (concentrated solution of SIF powder) in a concentration corresponding to the FeSSIF solution, pH increase, and an intensive agitation mimicking gastric emptying, formulations H and I released up to even 30% of labeled API dose within 30 min after the stress event. It indicated unwanted susceptibility to mechanical agitation in media simulating intestinal fluids. Similarly to fasted conditions, batch H was more prone to possible dose-dumping incidents. Batch J showed moderate resistance to mechanical agitation occurring during the simulated intragastric and intestinal passage. Upon obtained results, a reformulation was advised to decrease the sensitivity of the dosage form to mechanical agitation.

For the characterization of the prototypes under simulated fasted conditions, an online UV-VIS method was used to determine the concentration of the API in the media. In further experiments involving final batches, the signal of a UV detector showed unwanted characteristics, such as lack of repeatability or overestimation of the API amount in the media. After investigating the possible reasons, we concluded that the online UV-VIS method lacked the necessary discriminatory power to measure the API concentrations adequately. Therefore, for the characterization of the final batches, HPLC methods were applied, as described in Section 2.2.3.

The final batches contained 5, 30, and 120 mg of the API and were manufactured with two compression forces −16–17 kN, corresponding to the hardness of 140–175 N, and 22–23 kN, corresponding to the hardness of 185–225 N. First, all of the final batches were tested under standard protocols simulating both fasted and fed conditions. Figure 4C,D show the results of these experiments. All of the tested final batches had highly similar dissolution profiles, especially under simulated fasted conditions (Figure 4C). In addition, the variability of the profiles was lower than the prototype batches (Figure 4A). The variability was greater under simulated fed conditions, but the dissolution kinetics and the release rate of the API from the dosage forms were comparable regardless of the dose and the applied compression force (Figure 4D). Due to the better resistance and lower variability, the next step of the experiment involved batches compressed with a greater force.

**Figure 5:**
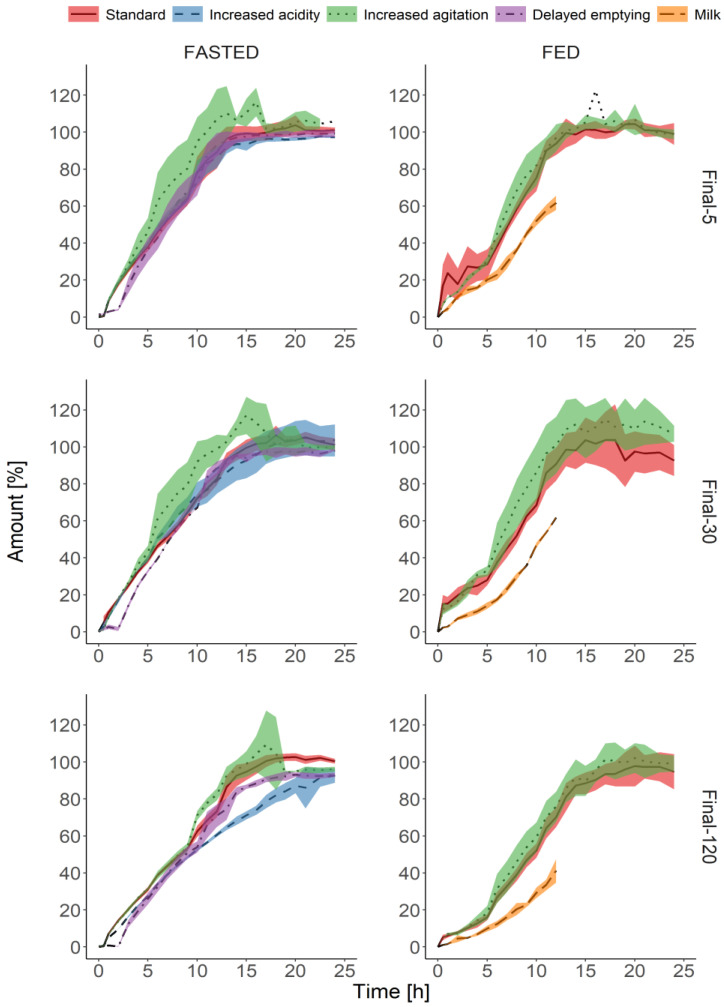
Comparison of dissolution profiles of the final formulations (hardness 185–225 N) containing 5, 30, and 120 mg API, obtained in biorelevant media in the StressTest device under simulated modified fasted and fed conditions. The data are presented as means of the cumulative amount of the drug released in percent (*n* = 6, *n* = 4 for the “Increased agitation” protocol under simulated fed conditions) with standard deviations presented as shaded areas.

Figure 5 shows the dissolution characteristics of the final batches (hardness 185–225 N) under modified biorelevant conditions, as described in Table 1. Under fasted conditions, the tested batches behaved similarly, and the dissolution profiles were aligned with the exception of the “Increased agitation” protocol. During this experimental setup, all tested batches, including the highest labeled API dose, were most prone to mechanical agitation. Interestingly, in the “Delayed gastric emptying” protocol, the batches showed the greatest resistance to the stress events. After the transition from the acidic media to the media simulating intestinal fluids, there were little or no noticeable increases in the cumulative amount of the dissolved API; this characteristic was most evident for 120 mg formulation. However, the formulation containing the highest labeled dose of 120 mg was also more resistant to mechanical agitation in comparison with low and medium-strength tablets.

Increased agitation during the experiment caused greater dissolution also under simulated fed conditions (Figure 5). Similarly to the fasted conditions, the 120 mg dose was the least prone to the simulated stress events. The dissolution kinetics differed to the greatest extent when the media consisted of milk with digestion enzymes. At each time point, the cumulative amount of the API was lower in comparison with “Standard” and “Increased agitation” protocols. The experiment conducted in milk took twelve hours only. Therefore, the prediction of the maximum cumulative amount released within 24 h was impossible.

## 4. Discussion

In the present study, we developed a robust and reliable SR dosage form of a novel GPR40 agonist for FIH pivotal clinical trial. We also introduce a framework for a comprehensive prediction of in vivo drug dissolution through physiology-oriented, biorelevant dissolution studies, standard USP dissolution, and characterization of mechanical properties of SR tablets. This novel approach has not been discussed yet in the available literature, especially in the context of the development of SR dosage forms with innovative medicines.

The European Medicines Agency (EMA) published the guidelines on strategies for FIH and early clinical trials [19]. EMA advises that several doses of the drug candidate should be evaluated in healthy volunteers, besides examination of non-clinical aspects, such as determination of strength and potency, use of a relevant animal model, evaluation of pharmacodynamics, pharmacokinetics, and toxicological properties. To maintain the dissolution characteristics over up to several dozen-fold dose increases, the preclinical formulation development has to rely on a good understanding of the physicochemical properties of the API [20]. In this process, the formulation specialists are crucial, as they can aid in obtaining early relative bioavailability information by proper design of the dosage forms used in the early stages of the trials and later commercialization ones [21]. They can also propose an optimal dosage form and choose the excipients that may alter API solubility, stability, or enhance bioavailability. At the formulation development stage, critical decision points include not only the development of the prototype and stability issues but a thorough evaluation of the reliability of its galenic principles under the application conditions [22]. Later stages of FIH trials may assess food and formulation effects [21]. Therefore, the preclinical characterization of the dosage form should also estimate its performance under both fasted and fed conditions. It minimizes the risk of an undesired performance in vivo, leading to failure of the trial and consequently delays and costs due to reformulation [23].

The aim of the discussed formulation development was to achieve a dosage form with a similar dissolution rate for all tablet strengths: 5, 30, and 120 mg. Due to the required prolonged in vivo exposure to the API, reaching 14–18 h after administration, we proposed an SR monolithic matrix tablet as the dosage form. The planned clinical trial protocol involved a dose-escalation step and estimation of the possible food effects. For this reason, the second objective was to evaluate the performance of the dosage form regardless of the prandial state. The third goal was to minimize the risk of undesired characteristics, such as dose dumping. Lastly, we wanted to assure comparable dissolution kinetics under altered physiological conditions, such as decreased or elevated gastric pH and increased GI motility.

In the first step, the standard dissolution tests allowed quick discrimination of the potential candidates (Figure 2A). At this stage, the matrix-filling and matrix-forming excipients were selected. As shown in Figure 2B, the USP-based standard dissolution tests allowed obtaining several candidate formulations of extended dissolution performance with a very small intra-batch variability. The dissolution from the prototypes H-K would ascertain a proper delivery within the required time frame. However, in the physiological conditions, the swollen tablet matrix is subjected to varied mechanical stress patterns and fluids of different compositions; these conditions change according to the prandial state. Therefore, we introduced more sophisticated dissolution tests that reflected the physiology of the GI tract to identify unwanted effects, such as dose dumping.

The StressTest device used in the present study allows the simultaneous simulation of the mechanical agitation in the GI tract and evaluation of dissolution in biorelevant media. Initially, the most promising prototypes selected in the USP dissolution tests were evaluated under standard fasted and fed conditions, as described in Table 1. The stress patterns and pH profiles were based on the data gathered in experiments using telemetric capsules [24,25,26]. The media for the fasted state were SGF *sine* pepsin pH = 1.8 and 50 mM potassium phosphate buffer with the addition of FaSSIF/FeSSIF/FaSSGF in concentrations relevant to the fasted conditions; for the fed state, the medium was 50 mM potassium phosphate buffer to which FaSSIF/FeSSIF/FaSSGF was added in concentration relevant to the fed state.

Usually, carbonate-based buffers, such as Hanks buffer, are preferred to simulate intestinal conditions in the fasted state. It is due to the fact that it better reflects the ionic composition and buffer capacity of small intestinal fluids [27]. In the present study, we initially used the Hanks buffer pH = 6.8 and adjusted to pH = 6.5 five hours after the beginning of the experiment. However, due to the undesired signal characteristics, the quantitative assessment of dissolution was unreliable between batches. This observation was non-formulation dependent and forced the transition from Hanks buffer to the higher capacity 50 mM phosphate buffer. For the same reason, an online UV-VIS measurement was changed to a high-throughput HPLC method, which was labeled as method ‘B’ in Section 2.2.3. A subsequent comparison of the dissolution characteristics in Hanks buffer versus 50 mM phosphate buffer revealed that the profiles were similar, but the tested tablets were more prone to mechanical agitation in the latter medium (data not shown). We concluded that the standard 50 mM potassium buffer was a medium of higher discriminatory power for the investigated formulations and adopted it in further experiments.

The application of physiologically relevant agitation during dissolution tests in the StressTest device revealed undesired characteristics of the tested prototypes (Figure 4A,B). The prototype tablets were prone to the applied pressure and mechanical agitation. Increased dissolution from the tablets was most pronounced under simulated fasted conditions. Although the dissolution kinetics of the prototypes were similar in the first hours of the experiment, a prolonged incubation in the medium and programmed stress events differentiated between the tested formulations. The bio-predictive dissolution tests revealed that the prototypes could release a significant amount of the API within a short time. These dose-dumping events could occur both under fasted and fed conditions. The accelerated release of API or dependability of the dosage form on mechanical stress can lead to increased pharmacokinetic variability [11,12]. Accurate estimation of pharmacokinetics is essential for innovative medicines under investigation. Therefore, a reliable dosage form with predictable dissolution kinetics and sufficient resistance to mechanical agitation in the GI tract is an asset in the planned clinical trial. This is the first study available for the public domain that shows the utilization of the StressTest device to evaluate properties of a dosage form for FIH clinical trials. Until now, the device has been successfully used to evaluate dissolution characteristics of generic SR or delayed-release products [28,29,30,31].

The composition of the prototypes was redesigned, and final batches were obtained. These batches were manufactured with two different compression forces. As a result, obtained were tablet batches with hardnesses of 140–175 N and 185–225 N, respectively. All the batches had outstanding robustness and integrity that was maintained regardless of the dose strength or the compression force used (Figure 2C and Figure 4C,D). Regardless of the hardness, the final batches of tablets were characterized by a similar dissolution performance under all applied test conditions. However, the batches manufactured at higher compression forces were more favorable for further technological processing such as dedusting, blistering, and even coating. Therefore, tablets of hardness 185–225 N were preferred to use in the clinical trial. The results of swelling experiments confirmed the robustness of the final formulations. The maximum incubation times in the swelling experiments (2 h for 0.1% HCl or 5 h for phosphate buffer and FaSSIF) were based on the observations from in vivo experiments with telemetric capsules [24,25,26]. The highest recorded fortitudes of the physiological stresses under both fasted (gastric emptying and ileocecal passage) and fed (gastric grinding and gastric emptying) occur within the first five hours after the intake. This time frame allowed us to estimate the performance of the formulation and the kinetics of the matrix-forming process. The test showed that the final formulations of hardness 185–225 N absorbed similar amounts of water and had comparable swelling kinetics, regardless of the API amount. It is noteworthy that a considerable area of the tablet core remained dry even after five hours of incubation in various media. It translated into an extended and regular release of the API within the desired 14–18 h time frame, as observed in the biorelevant dissolution tests. These results emphasize that simple and quick swelling experiments support and complement more complex analyses.

An advanced texture analysis also confirmed observations from the StressTest device (Figure 3). The resistance of the final batch tablets (hardness 185–225 N) to the deformation force was only slightly affected by the media. Although the tablet of the highest strength (120 mg) yielded the least to the applied force, the deformation study results were similar for all tested potencies.

The dissolution tests on final batches (hardness 185–225 N) comprised various test programs aimed at mimicking altered GI conditions (Figure 5). The tests should confirm or exclude the robustness observed under standard biorelevant protocols. For fasted conditions, we selected the conditions that emulated decreased pH of the gastric media, which could be observed in subjects with GI diseases, such as ulcers [32]. Another protocol evaluated the influence of a delayed gastric emptying time, such as in subjects with gastroparesis [33,34]. To ascertain sufficient mechanical integrity of the matrix tablets, one of the proposed protocols included prolonged mechanical agitation increased to the highest values of up to 350 mbar intensity. The increased agitation was also introduced in the test protocols under fed conditions during the intragastric stage and gastric emptying. Finally, the dissolution tests explored the possible impact of dynamically digested meals on the release of the API from the matrix tablets. Several media were suggested in the literature to simulate the composition and properties of GI fluids under the fed state. Ideally, the physicochemical properties and composition of the media should resemble a meal, such as standard breakfast in bioequivalence studies; it should also be adjustable to mirror the changes during the digestion [30]. One of the proposed is FeSSIF, which is used in the present study as a “standard” medium. Other alternatives are heat-treated whole milk and Ensure^®^ Plus. Another aspect is the digestion protocol. Two main approaches are dynamic digestion when enzymes such as pancreatin or lipase are added into the media [35] or the use of the “snapshot” media that reflect an early, middle, and late environment in the postprandial stomach [36,37]. In the present experiment, we used whole milk acidified with hydrochloric acid and digested it with pepsin and subsequently with pancreatin at elevated pH after simulated gastric emptying. We found that this medium is sufficiently stable and reflects well the physiological processes in the GI tract [35]. Of note, the duration of the dissolution experiments in milk was shorter than for the rest of the test setups (12 h vs. 24 h). There are several reasons that justify the selected time span. First, the test was designed to evaluate the resistance of the dosage form toward combined mechanical and physicochemical conditions that are representative of the stomach and small intestine in the postprandial state. Second, the digested milk is poorly stable during incubation at 37 °C, and the manual sampling and immediate sample processing require an extensive workload. Another aspect worth discussing is an incomplete dissolution of the API in these tests: 40–60% of the labeled amount within 12 h. The test simulates the worst-case scenario to which the dosage form can be subjected after the intake under fed conditions. Per definition, the dissolution of the tablet was not completed, and there was no plateau phase in the recorded profiles. As the API dissolved well in simulated gastric and intestinal conditions, the incomplete dissolution due to precipitation in the digested milk is unlikely.

We observed that in the tests performed under increased agitation protocol, the dissolution profiles differed to the greatest extent in comparison with the standard conditions. The variability was also greatest when the most intensive stress patterns were applied. The dissolution kinetics were comparable for all tested dose levels and test protocols. The only exception was dissolution experiments conducted in milk. In this medium, the amount of the API released and the rate of this process were the lowest. Before the experiment, we evaluated whether the API could be digested by the enzymes added into the media and confirmed that the molecule is resistant to such digestion. Therefore, the observed effect did not result from the instability of the API.

The decrease in the dissolution rate in milk is consistent with the results reported elsewhere [35,38,39]. Several hypotheses may explain such an observation. First, a proteinaceous film may form on the outside of the tablets from precipitates that increase tablet disintegration time [40]. This effect is most pronounced for immediate-release tablets; however, it is also likely to occur for the SR tablets. Second, due to the higher viscosity of milk than compendial media, the wetting of the tablet may be delayed [38]. Third, lipids may deposit on the outer gel layer of the matrix tablet that controls the rate of drug dissolution; these fat deposits may possess water barrier properties and prevent water from penetrating into the tablet core [39]. Milk also affects the solubility of drugs, such as dicumarol, danazol, or mefenamic acid [41,42]. It is most pronounced for BSC class II drugs. The API in the present study was classified as a BCS class I drug; therefore, its solubility should not be greatly affected by milk, and the observed dissolution variability is formulation-dependent.

The outcome of this study was SR formulations of ascending strength of a novel GPR-40 agonist. They allowed a regular drug delivery within 14–18 h, according to the clinical specification. The final formulations of 185–225 N hardness, which were selected for the clinical trial, showed very consistent and similar dissolution kinetics under both fasted and fed biorelevant conditions, regardless of the dose labeled. The tablets were also resistant to non-standard conditions in the GI tract, such as delayed gastric emptying, increased mechanical agitation, or low gastric pH. The results indicate that the developed formulations bear no risk of dose dumping and allow a predictable drug delivery, which is of great importance for antidiabetic drugs.

Lastly, the strength and novelty of the present study is the range and flexibility of the research protocol, connecting simple experiments with sophisticated dissolution analysis. This unique combination allows targeting specific requirements resulting from the physicochemical or pharmacokinetic properties of the API. Moreover, the biorelevant dissolution protocols specified in the StressTest device can be adopted for a wide range of GI conditions, such as simulation of the variability of the passage times, varied stress patterns, or evaluation of food effects. Customization of the conditions allows a swift reformulation and optimization of the dosage form before entering the clinical trials. Therefore, the implementation of the characterization strategy described in this paper into the development cycle of innovative medicines allows shortening the development times and reduces the risk of the FIH clinical trial failure, dose finding, and later therapy.

## 5. Conclusions

In this study, we showed a varied and thorough approach for the development and characterization of SR matrix tablets containing an innovative GPR40 agonist for use in the first-in-human clinical trial. The tests were performed using bio-predictive dissolution tests and a set of experiments determining the performance of the galenic principles of the formulations, such as texture and matrix formation analysis using texture analyzer and evaluation of the swelling performance. The formulations containing 5, 30, and 120 mg of the API developed according to implemented strategy were exceptionally robust toward the mechanical and physicochemical conditions of the bio-predictive tests and assured comparable drug delivery rates regardless of the prandial state and dose labeled. These characteristics were desired for the upcoming clinical trial. We conclude that the implementation of the development strategy described in this paper into the development cycle of SR formulations with innovative APIs helps not only to reduce the risk of formulation-related failure of phase I clinical trial but also to effectively and timely provide reliable medicines for patients in the trial and their further therapy.

## Figures and Tables

**Table 1:** Detailed setup of the test programs used in biorelevant dissolution tests.

Program		Intragastric Conditions	Gastric Emptying	Intestinal Passage	Ileocaecal Passage	Colonic Passage
Simulated fasted conditions						
Program 1 Regular gastric emptying	Time points Pressure Mech. agitation	0–0.5 h None No agitation	0.5 h 3 × 300 mbar 60 s × 100 rpm	1, 2 and 3 h 1 × 150 mbar 30 s × 50 rpm	5 h 2 × 200 mbar 30 s × 50 rpm	9, 12, 16 and 20 h 2 × 200 mbar 30 s × 50 rpm
	Medium	USP SGF sine pepsin, pH = 1.8		50 mM potassium phosphate buffer pH = 6.8 + 2.24 g/L FaSSIF/FeSSIF/FaSSGF		
Program 2 Acidified gastric environment	Time points Pressure Mech. agitation	0–0.5 h None No agitation	0.5 h 3 × 300 mbar 60 s × 100 rpm	1, 2 and 3 h 1 × 150 mbar 30 s × 50 rpm	5 h 2 × 200 mbar 30 s × 50 rpm	9, 12, 16 and 20 h 2 × 200 mbar 30 s × 50 rpm
	Medium	USP SGF sine pepsin, pH = 1.2		50 mM potassium phosphate buffer pH = 6.8 + 2.24 g/L FaSSIF/FeSSIF/FaSSGF		
Program 3 Increased mechanical agitation	Time points Pressure Mech. agitation	0–0.5 h None No agitation	0.5 h 3 × 350 mbar 80 s × 100 rpm	1, 2 and 3 h 1 × 300 mbar 30 s × 50 rpm	5 h 2 × 300 mbar 30 s × 50 rpm	9, 12, 16 and 20 h 2 × 300 mbar 30 s × 50 rpm
	Medium	USP SGF sine pepsin, pH = 1.8		50 mM potassium phosphate buffer pH = 6.8 + 2.24 g/L FaSSIF/FeSSIF/FaSSGF		
Program 4 Delayed gastric emptying	Time points Pressure Mech. agitation	0–2 h None No agitation	2 h 3 × 300 mbar 60 s × 100 rpm	2.5, 3.5, and 4.5 h 1 × 150 mbar 30 s × 50 rpm	6.5 h 2 × 200 mbar 30 s × 50 rpm	11.5, 13.5, 17.5, and 21.5 h 2 × 200 mbar 30 s × 50 rpm
	Medium	USP SGF sine pepsin, pH = 1.8		50 mM potassium phosphate buffer pH = 6.8 + 2.24 g/L FaSSIF/FeSSIF/FaSSGF		
Simulated fed conditions						
Program 5 Regular gastric emptying	Time points Pressure Mech. agitation	2, 3, and 4 h 2 × 200 mbar 30 s × 50 rpm	5 h 3 × 300 mbar 60 s × 100 rpm	6, 7, and 8 h 2 × 150 mbar 30 s × 50 rpm	10 h 2 × 200 mbar 60 s × 50 rpm	12, 16, and 20 h 3 × 200 mbar 30 s × 50 rpm
	Medium	50 mM potassium phosphate buffer 0–0.5 h: pH = 4.5; 0.5–1 h: pH = 3.5; 1–2 h: pH = 3.0; 2–5 h: pH = 2.0		Addition of 40 mL FaSSIF/FeSSIF/FaSSGF dissolved in 50 mM phosphate buffer up to the final concentration of 11.2 g/L, and adjustment of pH to 6.5		
Program 6 Regular gastric emptying in milk with addition of digestive enzymes	Time points Pressure Mech. agitation	2, 3, and 4 h 2 × 200 mbar 30 s × 50 rpm	5 h 3 × 300 mbar 60 s × 100 rpm	6, 7, and 8 h 2 × 150 mbar 30 s × 50 rpm	10 h 2 × 200 mbar 60 s × 50 rpm	12, 16, and 20 h 3 × 200 mbar 30 s × 50 rpm
	Medium	Whole milk (3.5% fat) 0–0.5 h: pH = 4.5; 0.5–1 h: pH = 3.5; 1–2 h: pH = 3.0; 2–5 h: pH = 2.0 Addition of 1 mL pepsin solution after 0, 0.5, 1 and 2 h up to the final concentration of 2.0 g/L		Addition of 40 mL of aqueous solution containing FaSSIF/FeSSIF/FaSSGF and pancreatin. Final concentration of FaSSIF/FeSSIF/FaSSGF was 11.2 g/L, and final concentration of pancreatin was 4.57 g/L. Adjustment of pH to 6.5		
Program 7 Increased gastric mechanical agitation	Time points Pressure Mech. agitation	2, 3, and 4 h 4 × 300 mbar 30 s × 50 rpm	5 h 4 × 350 mbar 80 s × 100 rpm	6, 7, and 8 h 2 × 150 mbar 30 s × 50 rpm	10 h 2 × 200 mbar 60 s × 50 rpm	12, 16, and 20 h 3 × 200 mbar 30 s × 50 rpm
	Medium	50 mM potassium phosphate buffer 0–0.5 h: pH = 4.5; 0.5–1 h: pH = 3.5; 1–2 h: pH = 3.0; 2–5 h: pH = 2.0		Addition of 40 mL of FaSSIF/FeSSIF/FaSSGF dissolved in 50 mM phosphate buffer up to the final concentration of 11.2 g/L, and adjustment of pH to 6.5		

FaSSIF/FeSSIF/FaSSGF—Fasted State Simulated Intestinal Fluid/Fed State Simulated Intestinal Fluid/Fasted State Simulated Gastric Fluid; SGF—simulated gastric fluid.

**Table 2:** Results of the water sorption and swelling kinetics analysis after incubation of tablets in different media. Water sorption analysis and increase of tablets’ height and width are presented as comparisons with the initial measurements. The dry areas are presented as percentages of the total area of the tablet cross-section after incubation in the medium (dry area/total area × 100%). Data are presented as means with standard deviations.

Medium		Formulation			
		J	Final-5	Final-30	Final-120
	Water sorption [%] (*n* = 3)				
0.1% HCl (2 h)		97.26 ± 3.82	94.91 ± 2.44	95.58 ± 1.24	95.80 ± 0.59
PP pH = 3.0 + 0.1% Tween (5 h)		205.79 ± 4.03	169.49 ± 4.55	179.44 ± 2.23	173.19 ± 3.58
FaSSIF (5 h)		199.19 ± 2.48	168.79 ± 0.62	161.59 ± 4.18	154.93 ± 0.16
	Swelling kinetics [%] (*n* = 3)				
0.1% HCl (2 h)	Height	184.33 ± 17.42	168.55 ± 5.30	179.94 ± 14.20	174.07 ± 16.38
	Width	152.60 ± 16.07	125.24 ± 5.82	133.06 ± 13.37	118.93 ± 2.95
	Dry area	26.68 ± 2.15	50.66 ± 3.25	50.50 ± 6.26	60.12 ± 4.61
PP pH = 3.0 + 0.1% Tween (5 h)	Height	204.44 ± 8.27	178.92 ± 17.64	214.64 ± 9.84	188.21 ± 13.48
	Width	145.01 ± 3.81	117.51 ± 15.78	127.09 ± 5.83	119.43 ± 10.72
	Dry area	33.50 ± 2.10	38.91 ± 7.18	43.14 ± 10.71	37.27 ± 10.41
FaSSIF (5 h)	Height	190.14 ± 2.89	179.43 ± 6.72	191.85 ± 11.31	179.43 ± 8.07
	Width	131.55 ± 3.45	117.98 ± 5.68	118.35 ± 14.94	118.42 ± 13.03
	Dry area	26.65 ± 2.34	45.27 ± 9.36	42.84 ± 2.81	38.53 ± 2.27

PP—50 mM phosphoric buffer, FaSSIF—Fasted State Simulated Intestinal Fluid.

## Data Availability

The data presented in this study are available on request from the corresponding author. The data are not publicly available due to an ongoing development and registration process.

## Supplementary Materials

The following are available online at https://www.mdpi.com/article/10.3390/pharmaceutics13060804/s1, Supplementary File containing Figure S1: A sample cross-section of a formulation after incubation in the test medium, as described in Section 2.2.2 of the manuscript. The photograph depicts the final formulation containing 120 mg API (Final-120) after 5 h incubation in FaSSIF. The addition of the colorant to the medium allowed visualization of the hydration front, and Table S1: Composition of developed formulations A-K and the final one. The values are presented as mass percentages of the total mass of the tablet.
