# Supplementary material for: Development and Bio-Predictive Evaluation of Biopharmaceutical Properties of Sustained-Release Tablets with a Novel GPR40 Agonist for a First-in-Human Clinical Trial
Source: Pharmaceutics. 2021 May 28;13(6):804. doi: 10.3390/pharmaceutics13060804 (PMC8227174; doi:10.3390/pharmaceutics13060804)
Supplement: Supplementary file 1 [file pharmaceutics-13-00804-s001.zip › pharmaceutics-1225603-supplementary.pdf]

pharmaceutics

MDPI

Supplementary Materials: Development and Bio-Predictive Evaluation of Biopharmaceutical Properties of Sustained-Release Tablets with a Novel GPR40 Agonist for a First-in-Human Clinical Trial

Ewelina Juszczyk, Kamil Kisło, Paweł Żero, Ewa Tratkiewicz, Maciej Wieczorek, Jadwiga Paszkowska, Grzegorz Banach, Marcela Wiater, Dagmara Hoc, Grzegorz Garbacz, Jaroslaw Sczodrok and Dorota Danielak

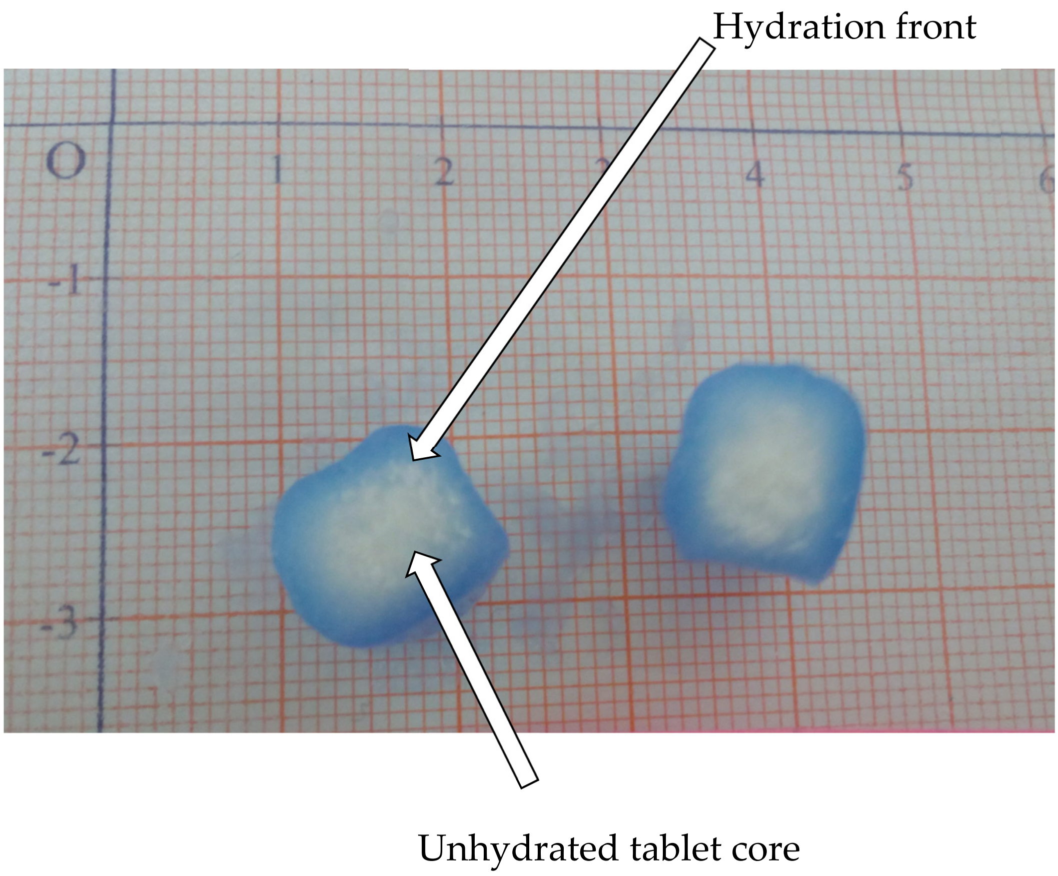

Figure S1. A sample cross-section of a formulation after incubation in the test medium, as described in Section 2.2.2 of the manuscript. The photograph depicts the final formulation containing 120 mg API (Final-120) after 5 h incubation in FaSSIF. The addition of the colorant to the medium allowed visualization of the hydration front.

Table S1. Composition of developed formulations A-K and the final one. The values are presented as mass percentages of the total mass of the tablet.

Ingredient	Ingredient content in formulation batch [%]											
	A	B	C	D	E	F	G	H	I	J	K	Final
Hypromellose	20–30	10–20	10–20		5–10	20–30	5–15					
Silicified hypromellose								25–35	30–35	25–35	25– 35	25–35
Glycerol dibehenate				15–25								
Microcrystalline cellulose	5–10	20–30		5–15	25–35	10–15	20–30					
Pregelatinized starch								25–35	25–35	35–40	40–45	35–40
Mannitol			20–30									
Lactose monohydrate								5–10	5–10	5–10	5–10	5–10
Sodium hydrogen phosphate								5–10	5–10	5–10	5–10	5–10
Colloidal silica								< 2	< 2	< 2	< 2	< 2
Magnesium stearate	< 2	< 2	< 2	< 2	< 2	< 2	< 2	< 2	< 2	< 2	< 2	< 2
